# Medical, welfare, and educational challenges and psychological distress in parents caring for an individual with 22q11.2 deletion syndrome: A cross‐sectional survey in Japan

**DOI:** 10.1002/ajmg.a.62485

**Published:** 2021-09-03

**Authors:** Ryo Morishima, Yousuke Kumakura, Satoshi Usami, Akiko Kanehara, Miho Tanaka, Noriko Okochi, Naomi Nakajima, Junko Hamada, Tomoko Ogawa, Shuntaro Ando, Hidetaka Tamune, Mutsumi Nakahara, Seiichiro Jinde, Yukiko Kano, Kyoko Tanaka, Yoichiro Hirata, Akira Oka, Kiyoto Kasai

**Affiliations:** ^1^ Department of Neuropsychiatry, Graduate School of Medicine The University of Tokyo Tokyo Japan; ^2^ The Health Care Science Institute Tokyo Japan; ^3^ Department of Mental Health, Graduate School of Medicine The University of Tokyo Tokyo Japan; ^4^ The Graduate School of Education University of Tokyo Tokyo Japan; ^5^ Department of Child Psychiatry University of Tokyo Hospital Tokyo Japan; ^6^ Graduate School of Clinical Psychology Kagoshima University Kagoshima Japan; ^7^ Department of Child Neuropsychiatry, Graduate School of Medicine The University of Tokyo Tokyo Japan; ^8^ Division of Pediatric Consultation Liaison, Department of Psychosocial Medicine National Center for Child Health and Development Tokyo Japan; ^9^ Department of Pediatrics, Graduate School of Medicine The University of Tokyo Tokyo Japan; ^10^ The International Research Center for Neurointelligence (WPI‐IRCN) The University of Tokyo Institutes for Advanced Study (UTIAS) Tokyo Japan

**Keywords:** 22q11.2 deletion syndrome, educational challenges, medical challenges, parental psychological distress, welfare challenges

## Abstract

Parents of children with 22q11.2 deletion syndrome (22q11DS) experience distress not only due to multimorbidity in the patients, but also due to professionals' lack of understanding about 22q11DS and insufficient support systems. This study investigated relationships between medical, welfare, and educational challenges and parental psychological distress. A cross‐sectional survey was conducted on primary caregivers of children with 22q11DS. Participants included 125 parents (114 mothers, 91.2%; average age = 44.3 years) who reported their challenges, psychological distress, and child's comorbidities of 22q11DS. Results showed that the difficulty in going to multiple medical institutions (*β* = 0.181, *p* < 0.05) and lack of understanding by welfare staff and insufficient welfare support systems for 22q11DS (*β* = 0.220–0.316, all *p* < 0.05) were associated with parental psychological distress, even after adjusting for child's comorbidities. In the subsample of parents whose child attended an educational institution, inadequate management in classroom and mismatch between service and users in educational settings were associated with psychological distress (*β* = 0.222–0.296, all *p* < 0.05). This study reveals the importance of assessing not only severity of comorbidities in 22q11DS, but also the medical, welfare, and educational challenges for parental mental health.

## 1 INTRODUCTION

22q11.2 deletion syndrome (22q11DS) is the most common microdeletion syndrome with an estimated prevalence of 1:2000–1:6000 live births (McDonald‐McGinn, 2018). Individuals with 22q11DS experience multiple physical, developmental, and psychiatric chronic conditions (multimorbidity) throughout their life‐span, resulting in an increased burden on their caregivers (Cywińska‐Bernas et al., 2018; Hercher & Bruenner, 2008; Karas et al., 2014; Reilly et al., 2015). These conditions, including behavioral problems, may be associated with increased parental stress (Briegel et al., 2007, 2008). Approximately one of three to seven parents reported restrictions in daily activities due to caring for an individual with 22q11DS (Reilly et al., 2015). Parents having a child with 22q11DS had higher future concerns such as general health and academic achievement for their child than those whose child had other neurogenetic syndromes (Reilly et al., 2015). This may be due to high prevalence of psychiatric disorders and difficulties in cognitive performance and daily living skills (Mosheva et al., 2019). Parents perceived themselves as trapped in a lifelong career of care (Goodwin et al., 2017). These findings suggest the need for establishing support systems to reduce the burden on individuals with 22q11DS and their families.

However, there is a scarcity of research exploring the relationship between professionals' lack of understanding about 22q11DS and paucity of support systems and psychological distress in parents caring for individuals with 22q11DS. A few qualitative studies have indicated the relationship between medical, welfare, and educational challenges and distress in the family (Bales et al., 2010; Goodwin et al., 2017; Klingberg et al., 2010; Vo et al., 2018). A previous cross‐sectional survey on 34 parents and caregivers reported several difficulties faced by children with 22q11DS at school, including curricular and/or general aspects (Cohen et al., 2017). Moreover, a web‐based survey on 274 parents caring for children with rare diseases suggested that issues such as health professionals' lack of understanding about the condition and lack of support systems may lead to parental dissatisfaction and distress (Pelentsov et al., 2016). However, these studies either were qualitative, had a small sample size, or comprised a heterogeneous population. Parents caring for an individual with 22q11DS have expressed the need for further research into developing and improving psychosocial support as well as physical medicine (Tamune et al., 2020). Evaluating the effects of medical, welfare, and educational challenges on parental psychological distress, using a large sample of families caring for an individual with 22q11DS, can provide practical implications and useful directions to establish a comprehensive support system for individuals with 22q11DS and their families.

This study aimed to evaluate the relationship between medical, welfare, and educational challenges and psychological distress in parents of individuals with 22q11DS. We hypothesized that medical, welfare, and educational challenges would be associated with increased psychological distress in parents, even after adjusting for the multimorbidity in their child. Especially, professionals' lack of understanding about 22q11DS and lack of support systems in medical, welfare, and educational areas would be related to parental psychological distress.

## 2 METHODS

### 2.1 Editorial policies and ethical considerations

This study was approved by the Ethical Committee of the Faculty of Medicine at the University of Tokyo [approval no. 2018015NI‐(7)]. On the first page of the web‐based survey and on the cover page of the paper‐based survey, participants were instructed about the overall purpose of the study. It was also explained that informed consent was obtained through the participant's response to the survey. A response to this survey was restricted to once per family.

### 2.2 Target population

We conducted a web‐based and paper‐based survey on medical, welfare, and educational support needs and challenges experienced by primary caregivers of individuals with 22q11DS. The survey was conducted from March 20 to November 8, 2019. The designated responders were primary caregivers, such as mother or father, of children with 22q11DS or children suspected of having 22q11DS but without a formal diagnosis.

An entrance of the web questionnaire was set up on our research team web page (“22q‐pedia”; https://22q-pedia.net/). The survey was publicized to the patients' families by e‐mail and/or letter with the help of the Japanese 22q11DS family associations, family associations of patients with pediatric heart disease, and people involved in the medical care of 22q11DS. (Therefore, the recruited population was unclear, and the collection rate could not be calculated.) For participants who found it difficult to answer the web questionnaire, the paper questionnaire was sent to them by mail upon request. Of the 125 valid responses, four were via the paper questionnaire.

### 2.3 Development of the survey

We developed the items of the survey based on previous literature and clinical guidance (e.g., Basset et al., 2011; Campbell et al., 2018; Fung et al., 2015; Habel et al., 2014) with the assistance of two parents caring for an individual with 22q11DS. After developing the first draft of the questionnaire, the validity of the items was assessed by clinical and research experts (M.F., Y. Kano, Y.H., S.Y., Y.M., and S.A.) and the assisting parents. The aforementioned clinical and research experts either worked in the field of rare diseases or had survey development expertise. Invalid items identified by these experts and parents were modified or excluded by an advisor of family meetings (K.K.) and an outpatient clinician for 22q11DS (Y. Kumakura).

The final version of the survey questionnaire was divided into two main parts (Part A and Part B), which were subdivided into 10 domains, as follows: (1) Demographics about parents (Part A); (2) Demographics and child's diagnosis of comorbidities in an individual with 22q11DS (Part A); (3) Parental psychological distress and various challenges in medical, welfare, educational, and other areas (Part A); (4) Situation of notification of 22q11DS diagnosis, related impact, and required support in medical areas (Part B); (5) Information about disability certificates and required support in welfare areas (Part B); (6) Information about educational attainment and required support in educational areas (Part B); (7) Problems related to transition from pediatric to adult medical care (Part B); (8) Sibling issues (Part B); (9) Research needs (Part B); and (10) Positive and negative changes in parents' lives (Part B). Items in Part A were mandatory, whereas those in Part B were optional.

### 2.4 Independent and dependent variables and covariates

For this study, we designated parental psychological distress as the dependent variable and medical, welfare, and educational challenges as independent variables, from Domain 3 of the survey. Psychological distress was measured by the Kessler 6 (K6) scale, a six‐item screening measure for nonspecific psychological distress during the past 30 days (Kessler et al., 2002). The participants rated the items (e.g., “During the past 30 days, about how often did you feel so depressed that nothing could cheer you up?”) on a five‐point Likert‐type scale ranging from “none of the time” (0) to “all of the time” (4). The final score was calculated by summing up all the responses, with a possible range of 0–24. Higher scores indicated more severe psychological distress. The internal consistency (Cronbach's *α*) of the K6 in this study was 0.87.

Medical, welfare, and educational challenges in the past year were assessed using 14, 26, and 26 items, respectively (see Table S1). These challenges were assessed by the following question: “What challenges are you currently facing (in the past year) in terms of medical care/welfare/education as you support a family member with 22q11.2 deletion syndrome? Select all that apply.” The participants responded to each option (e.g., lack of information about 22q11DS) in a dichotomous format. We used the total number of items and each dichotomous item as independent variables. In line with the Japanese social welfare systems, we included items related to mainly administrative, social security, and development‐related social support services as welfare challenges (Table S1). In Japan, special education services through the public school system are included in the education system, which is described later. The internal consistencies (Cronbach's *α*) of the total number of medical, welfare, and educational challenges were 0.55, 0.83, and 0.78, respectively.

As covariates, we used the data about parental age, parental sex, annual household income, and marital status in Domain 1, and child's age, child's sex, and child's comorbidities in Domain 2. Child's comorbidities were assessed by the following question: “Select all diseases diagnosed from birth to the present. If you do not know which category the disease falls under, enter your response under ‘Other’. Do not include names of ‘suspected’ diseases.” The categories of child's comorbidities and options are shown in Table 1. All the options had a dichotomous response choice, except the last one. The “Other diseases” category was to be answered in an open‐ended format, and we considered the presence of other diseases if the participants provided the name/s of any disease/s under this option. We coded the response as “1” if the participants reported positive (Yes) for at least one option in each category, and otherwise as “0,” and determined the total score for the total number of child's comorbidities with a possible range of 0–9.

**TABLE 1:** List of child's comorbidities

Congenital heart disease
Tetralogy of Fallot, pulmonary atresia with ventricular septal defect (extreme tetralogy of Fallot), ventricular septal defect, atrial septal defect, aortic arch interruption, truncus arteriosus, and other congenital heart diseases
Immune system disorder
Immunodeficiency, repeated infection, and other immune/allergic disorders (e.g., atopic dermatitis, asthma, hay fever, etc.)
Endocrine disorder
Hypoparathyroidism, hypocalcemia, thyroid dysfunction, and other endocrine disorders
Gastrointestinal disease
Dysphagia, tube feeding, inguinal hernia, and other gastrointestinal diseases
Otorhinolaryngology/maxillofacial disease
Ear infection, hearing loss, cleft palate, cleft lip, nasopharyngeal incompetence, submucous cleft palate, and other otorhinolaryngology/maxillofacial diseases
Orthopedic disease
Scoliosis, pes valgus, clubfoot, cervical spine abnormality, and other orthopedic disorders
Growth/developmental disorder
Intellectual disability, autism spectrum disorder (ASD, Asperger's syndrome), attention deficit hyperactivity disorder, oppositional defiant disorder, learning disorder, speech delay, selective mutism, delayed motor development, growth disorder/short stature, and other growth/developmental disorders
Psychiatric/neurological disorder
Schizophrenia, depression, bipolar disorder (manic depression), anxiety disorder, panic disorder, obsessive–compulsive disorder, epilepsy, Parkinson's disease, and other neuropsychiatric disorders
Other diseases

### 2.5 Statistical analysis

Descriptive statistics were calculated for all variables. Pearson's correlation coefficient was computed to evaluate the association between the total number of child's comorbidities and parental psychological distress. Parental psychological distress was compared using analysis of variance (ANOVA) to evaluate differences between age intervals (infant to preschool: 0–5 years, school age: 6–12 years, adolescents and adults: 13 years or older). We also performed a *t*‐test to examine differences in parental psychological distress between children with each psychiatric/neurological disorder and those without.

The relationship between medical, welfare, and educational challenges and parental psychological distress was examined using hierarchical multivariate regression analysis. We performed three steps as follows: (1) Crude model; (2) adjusted Model 1: adjusted for parental age, parental sex, annual household income, marital status, child's age, and child's sex; and (3) adjusted Model 2: adjusted for the variables in Model 1 and the total number of child's comorbidities. Covariates and the total number as well as each item of the challenges that had a statistically significant association in the previous model were added simultaneously to the next model.

The effects of educational challenges on parental psychological distress may be observed specifically for children who attended an educational institution. Therefore, we conducted further hierarchical multivariable regression analysis using a subsample of parents whose children attended an educational institution and were aged 18 years or less. This definition was in line with Japanese educational systems. In Japan, before starting school, there are certain types of preschool educational institutions such as daycare (“*hoikuen*”; for children aged 0 years or more) or kindergarten (“*youchien*”; for children aged 3 years or more), and families can decide whether their child attends one or not. Compulsory educational institutions for children aged 6–15 years are classified broadly as elementary schools (6–12 years old), junior high schools (12–15 years old), or schools for special needs education (elementary department and junior high school department). Senior high school is for children who have completed the elementary and junior high schools and is normally completed in 3 years (15–18 years old). For children with disabilities, there are schools for special needs education with a senior high school department. If the participants did not respond to a dichotomous screening question in Domain 3 inquiring whether the child was enrolled in any preschool educational institution, or had a child older than 18 years, we excluded their data from this subsample. Finally, 84 participants (children's age range: 2–18 years) were eligible for the subsample.

All statistical analyses were conducted using the statistical software R, version 3.6.1 (the R Foundation for Statistical Computing, Vienna, Austria). A significance level was set to *α* = 0.05 for all analyses.

## 3 RESULTS

**FIGURE 1:**
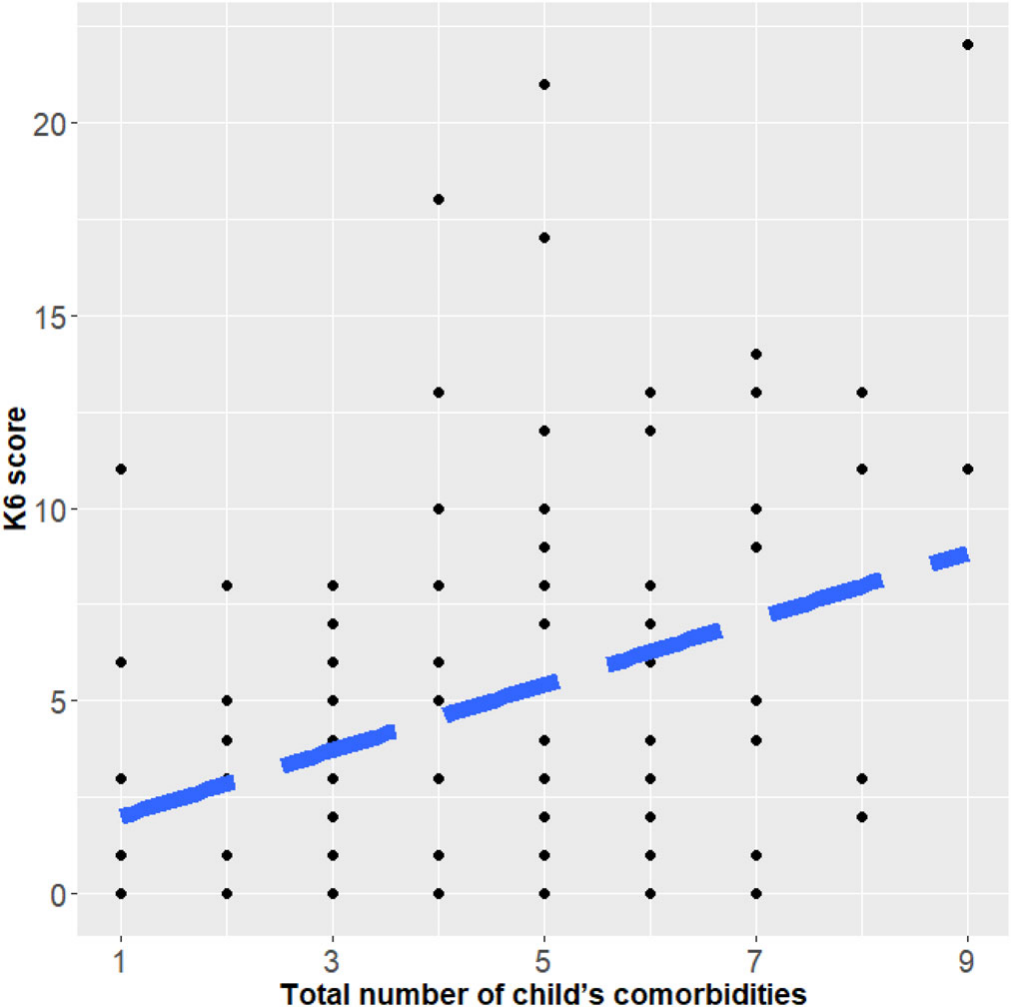
Correlation between total number of child's comorbidities and K6 score. K6, Kessler 6; dashed line represents regression line (*r* = 0.322, *p* < 0.001)

Descriptive statistics of variables are shown in Table 2. All the participants were parents (either mother or father) of individuals with 22q11DS. The mean age of participants was 44.3 years (standard deviation, *SD* = 7.5), and 91.2% of them were mothers. The mean age of individuals with 22q11DS was 11.8 years (*SD* = 7.7; range of 0–38 years), and about half of them were male. On average, individuals with 22q11DS had four or more lifetime comorbidities. The mean score of the K6 (psychological distress) in parents was 5.0 (*SD* = 4.6). The total number of child's comorbidities was associated with increased parental psychological distress (*r* = 0.322, *p* < 0.001; Figure 1). In the ANOVA, the main effect of age intervals was not significant (*F*_(2,122)_ = 0.204, *p* = 0.816), and therefore, we did not perform a post‐hoc test. In the *t*‐test, a positive diagnosis of schizophrenia and anxiety disorder in the child were associated with higher parental psychological distress (Table S2).

**TABLE 2:** Descriptive statistics of the study participants (total *N* = 125)

Parent	
Age, mean (*SD*)	44.3 (7.5)
Mother, *N* (%)	114 (91.2)
Father, *N* (%)	11 (8.8)
Annual household income, *N* (%)	
0–2.99 million yen	13 (10.4)
3–5.99 million yen	36 (28.8)
6–899 million yen	42 (33.6)
9–11.99 million yen	11 (8.8)
12–14.99 million yen	13 (10.4)
15–17.99 million yen	1 (0.8)
18 million yen or more	6 (4.8)
Unknown	3 (2.4)
Marital status, *N* (%)	
Single	3 (2.4)
Married	106 (84.8)
Divorced	13 (10.4)
Widowed	3 (2.4)
Kessler 6, mean (*SD*)	5.0 (4.6)
Individual with 22q11.2 deletion syndrome	
Age, mean (*SD*)	11.8 (7.7)
Male sex, *N* (%)	63 (50.4)
Lifetime comorbidities, mean (*SD*)	4.4 (1.8)
Lifetime comorbidities, *N* (%)	
Congenital heart disease	106 (84.8)
Immune system disorder	38 (30.4)
Endocrine disorder	48 (38.4)
Gastrointestinal disease	36 (28.8)
Otorhinolaryngology/maxillofacial disease	94 (75.2)
Orthopedic disease	51 (40.8)
Growth/developmental disorder	112 (89.6)
Psychiatric/neurological disorder	35 (28.0)
Other	24 (19.2)

The challenges with high frequency did not necessarily match the challenges related to parental psychological distress. The challenges with the highest frequencies were the following: “Lack of information regarding 22q11.2 deletion syndrome” (61.6%; in medical), “Lack of knowledge on the part of medical staff (doctors, nurses, etc.) regarding 22q11.2 deletion syndrome” (48.0%; in medical), “Lack of knowledge on the part of supporters (welfare facility personnel and government personnel) regarding 22q11.2 deletion syndrome” (48.0%; in welfare), “Lack of information regarding development‐related support such as rehabilitation” (44.0%; in welfare), and “Lack of knowledge regarding 22q11.2 deletion syndrome on the part of school staff (faculty, etc.)” (32.8%; in education) (Tables 3 and [Link], [Link]).

**TABLE 3:** Summary of hierarchical multivariate regression analysis on relationship between medical, welfare, and educational challenges and parental psychological distress (*N* = 125)

		Adjusted Model 1				Adjusted Model 2			
	Yes		95% CI				95% CI		
	*N* (%)	*β*	Lower	Upper	*p*	*β*	Lower	Upper	*p*
Medical challenges									
Total number of medical challenges, mean (*SD*)	*2.8 (2.0)*	**0.246**	**0.074**	**0.419**	**0.006**	**0.195**	**0.013**	**0.377**	**0.035**
Difficulty of going to multiple medical institutions	32 (25.6)	**0.214**	**0.046**	**0.382**	**0.013**	**0.180**	**0.012**	**0.349**	**0.036**
Welfare challenges									
Total number of welfare challenges, mean (*SD*)	*5.3 (4.4)*	**0.298**	**0.125**	**0.471**	**0.001**	**0.264**	**0.090**	**0.438**	**0.003**
Lack of consultants and contacts regarding financial support systems	25 (20.0)	**0.251**	**0.081**	**0.421**	**0.004**	**0.237**	**0.069**	**0.404**	**0.006**
Lack of knowledge on the part of supporters (welfare facility personnel and government personnel) regarding 22q11.2 deletion syndrome	60 (48.0)	**0.284**	**0.113**	**0.455**	**0.001**	**0.259**	**0.089**	**0.429**	**0.003**
No daycare center suitable for the characteristics and traits of the individual with 22q11.2 deletion syndrome	31 (24.8)	**0.294**	**0.124**	**0.465**	**0.001**	**0.268**	**0.098**	**0.438**	**0.002**
Unable to go to the daycare center even if one is available	12 (9.6)	**0.203**	**0.026**	**0.380**	**0.025**	0.162	−0.017	0.341	0.075
No support available when parents are exhausted and need a respite	25 (20.0)	**0.352**	**0.191**	**0.514**	**0.000**	**0.322**	**0.159**	**0.486**	**0.000**
Lack of understanding in the workplace	6 (4.8)	**0.264**	**0.089**	**0.439**	**0.003**	**0.239**	**0.065**	**0.413**	**0.008**
Lack of residences such as group homes	14 (11.2)	**0.194**	**0.005**	**0.384**	**0.045**	0.161	−0.028	0.350	0.094

*Note*: Bold represents statistically significant. Adjusted Model 1: multivariate regression analysis adjusting parental age, parental sex, family income, marital status, child age, and child sex. Adjusted Model 2: multivariate regression analysis adjusting parental age, parental sex, family income, marital status, child age, child sex, and total number of child's comorbidities.

Abbreviations: *β*, standardized regression coefficient; CI, confidence interval.

The challenges significantly related to parental psychological distress were as follows: “Difficulty of going to multiple medical institutions” (*β* = 0.180; in medical), “No support available when parents are exhausted and need a respite” (*β* = 0.322; in welfare), and “No daycare center suitable for the characteristics and traits of the individual with 22q11.2 deletion syndrome” (*β* = 0.268; in welfare) (Table 3). Educational challenges were not related to psychological distress (Table S5). The total number of medical (*β* = 0.195) and welfare challenges (*β* = 0.264) was also significantly associated with parental psychological distress (Table 3).

Further analyses were conducted using a subsample of parents whose child attended an educational institution (*N* = 84; Tables 4 and [Link], [Link]). The educational challenges with the highest percentage were the following: “Lack of knowledge regarding 22q11.2 deletion syndrome on the part of school staff (faculty, etc.)” (40.5%), “Lack of understanding on the part of school staff (faculty, etc.) of the fact that children with 22q11.2 deletion syndrome may need more individual attention than children with ordinary physical or intellectual disabilities” (32.1%), and “Lack of information regarding school selection” (22.6%) (Tables 4 and S8). The educational challenges strongly related to parental psychological distress were as follows: “Participating in extracurricular lessons and activities” (*β* = 0.338), “Refusal to attend school” (*β* = 0.292), and “The regular class teachers were unhelpful” (*β* = 0.292). The total number of educational challenges was significantly associated with parental psychological distress (*β* = 0.269; Table 4).

**TABLE 4:** Summary of hierarchical multivariate regression analysis on relationship between medical, welfare, and educational challenges and parental psychological distress among subsamples (*N* = 84)

		Adjusted Model 1				Adjusted Model 2			
	Yes		95% CI				95% CI		
	*N* (%)	*β*	Lower	Upper	*p*	*β*	Lower	Upper	*p*
Medical challenges									
Difficulty of going to multiple medical institutions	23 (24.0)	**0.293**	**0.073**	**0.514**	**0.010**	**0.273**	**0.048**	**0.499**	**0.018**
Welfare challenges									
Total number of welfare challenges, mean (*SD*)	*4.8 (4.1)*	**0.335**	**0.110**	**0.559**	**0.004**	**0.323**	**0.099**	**0.548**	**0.005**
Lack of consultants and contacts regarding financial support systems	20 (20.8)	**0.322**	**0.103**	**0.541**	**0.005**	**0.339**	**0.121**	**0.556**	**0.003**
Lack of consultants and contacts regarding development‐related support such as rehabilitation	30 (31.2)	**0.247**	**0.020**	**0.473**	**0.033**	**0.245**	**0.020**	**0.470**	**0.033**
Lack of knowledge on the part of supporters (welfare facility personnel and government personnel) regarding 22q11.2 deletion syndrome	40 (41.7)	**0.289**	**0.067**	**0.510**	**0.011**	**0.280**	**0.058**	**0.501**	**0.014**
No daycare center suitable for the characteristics and traits of the individual with 22q11.2 deletion syndrome	20 (20.8)	**0.394**	**0.175**	**0.613**	**0.001**	**0.378**	**0.156**	**0.601**	**0.001**
Unable to go to the daycare center even if one is available	8 (8.3)	**0.246**	**0.023**	**0.469**	**0.031**	**0.234**	**0.010**	**0.457**	**0.041**
Lack of home care/visiting services	7 (7.3)	**0.356**	**0.130**	**0.582**	**0.002**	**0.338**	**0.108**	**0.568**	**0.005**
No support available when parents are exhausted and need a respite	19 (19.8)	**0.498**	**0.294**	**0.701**	**0.000**	**0.487**	**0.283**	**0.691**	**0.000**
Educational challenges									
Total number of educational challenges, mean (*SD*)	*3.3 (3.1)*	**0.288**	**0.066**	**0.510**	**0.012**	**0.269**	**0.043**	**0.495**	**0.020**
No educational institutions suitable for the individual's characteristics/traits	19 (19.8)	**0.239**	**0.014**	**0.465**	**0.038**	**0.227**	**0.001**	**0.453**	**0.049**
The regular class teachers were unhelpful	6 (6.2)	**0.303**	**0.085**	**0.521**	**0.007**	**0.292**	**0.074**	**0.510**	**0.009**
The special class teachers were unhelpful	7 (7.3)	**0.287**	**0.058**	**0.515**	**0.015**	**0.270**	**0.040**	**0.500**	**0.022**
Refusal to attend school	5 (5.2)	**0.308**	**0.072**	**0.544**	**0.011**	**0.292**	**0.054**	**0.530**	**0.017**
Participating in extracurricular lessons and activities	9 (9.4)	**0.345**	**0.128**	**0.562**	**0.002**	**0.338**	**0.122**	**0.555**	**0.003**
Communicating with the home room teacher	9 (9.4)	**0.283**	**0.057**	**0.508**	**0.015**	**0.261**	**0.030**	**0.492**	**0.027**
Change of home room teacher	6 (6.2)	**0.307**	**0.085**	**0.529**	**0.007**	**0.289**	**0.050**	**0.528**	**0.019**

*Note*: Bold represents statistically significant. Adjusted Model 1: multivariate regression analysis adjusting parental age, parental sex, family income, marital status, child age, and child sex. Adjusted Model 2: multivariate regression analysis adjusting parental age, parental sex, family income, marital status, child age, child sex, and total number of child's comorbidities.

Abbreviations: *β*, standardized regression coefficient; CI, confidence interval.

## 4 DISCUSSION

To the best of our knowledge, this is the first large‐scale study to indicate the relationships between medical, welfare, and educational challenges and psychological distress in parents of an individual with 22q11DS, controlling for the total number of child's comorbidities. The difficulty of going to multiple medical institutions, lack of understanding by welfare staff, and lack of welfare support systems for 22q11DS were associated with parental psychological distress. In the subsample of parents whose child attended an educational institution, inadequate management in classroom and mismatch between service and users in educational settings were related to psychological distress in parents. A higher total number of medical and welfare challenges, and educational challenges in the subsample were associated with increased parental psychological distress.

The item “Difficulty in going to multiple medical institutions” was found to be associated with increased parental psychological stress, even after adjusting for the effect of multimorbidity in their child; one in four parents experienced this medical challenge. In many cases, parents needed to coordinate transportation for their child with 22q11DS to go to multiple medical institutions. This may have led to increased stress among parents. A previous qualitative study suggested that parents are stressed because of poor medical management (Bales et al., 2010). Medical difficulties were reported more often by parents of children with 22q11DS compared to those of children with other neurogenetic syndromes such as Fragile X syndrome, Prader Willi syndrome, and Williams syndrome (Reilly et al., 2015). Medical difficulties including coordinating transportation to go to multiple medical institutions might be more of an issue for families caring for a child with 22q11DS than for families caring for a child with other neurogenetic syndromes.

Regarding welfare challenges, the items “Lack of consultants and contacts regarding financial support systems,” “Lack of knowledge on the part of supporters (welfare facility personnel and government personnel) regarding 22q11.2 deletion syndrome,” “No daycare center suitable for the characteristics and traits of the individual with 22q11.2 deletion syndrome,” “No support available when parents are exhausted and need a respite,” and “Lack of understanding in the workplace” were associated with parental psychological distress. These challenges were reported by about 5%–50% of the participants. These findings are consistent with those of a previous qualitative study, indicating the negative emotional response in parents due to lack of understanding and adequate care for an individual with 22q11DS on the part of healthcare professionals (Goodwin et al., 2017). Since welfare services may be closely related to daily life in children with 22q11DS, a lack of understanding among welfare staff and any place where the child belongs such as daycare or workplace may influence parental mental health. Also, challenges related to welfare systems (e.g., financial support or respite care) may lead to distress in parents. This is consistent with the results of a previous web‐based survey on parents caring for a child with a rare disease, indicating an association between financial distress and parents' dissatisfaction toward received services (Pelentsov et al., 2016). Reducing the burdens (e.g., financial problems, lack of respite care) on parents caring for an individual with 22q11DS may improve their mental health. Furthermore, the effects of “Lack of residences such as group homes” and “Unable to go to the daycare center even if one is available” on parental psychological distress were explained by the total number of child's comorbidities (Table 3). The existing social system (especially in welfare) was found to be insufficient to enable individuals with multimorbidity to live or work independently, leading to an increased burden on the family due to the necessity for lifelong care of these individuals (Goodwin et al., 2017). A previous study reported that functional independence, such as using telephone, using public transportation, and attending social events, is associated with better mental health in mothers of children with other neurogenetic condition such as Down syndrome (e.g., Bourke et al., 2008). Social systems that could support the independence of individuals with 22q11DS and those with other neurogenetic conditions might improve parental mental health, but this possibility needs further research.

In the parents whose child attended an educational institution and was aged 18 years or less, educational challenges such as “No educational institutions suitable for the individual's characteristics/traits,” “The regular/special class teachers were unhelpful,” “Refusal to attend school,” “Participating in extracurricular lessons and activities,” and “Communicating with/change of the home room teacher” increased parental psychological distress, even after adjusting for covariates (Table 4). These challenges were experienced by about 5%–20% of parents in the subsample. A previous cross‐sectional survey indicated the difficulties encountered at schools, such as lack of understanding of 22q11DS among teachers, school absence due to illness, and difficulties in following instructions (Cohen et al., 2017). This study added that these educational challenges within the school environment (e.g., “The regular/special class teachers were unhelpful,” “Refusal to attend school,” and “Communicating with/change of the home room teacher”) may be associated with increased distress in parents. Lack of progress at school was a common challenge for some children with neurogenetic syndromes including 22q11DS (Reilly et al., 2015). Therefore, solving educational challenges might improve the mental health of parents caring for children with 22q11DS and other neurogenetic syndromes. Moreover, educational systems and extracurricular activities outside the school (e.g., “No educational institutions suitable for the individual's characteristics/traits” and “Participating in extracurricular lessons and activities”) may also be related to parental psychological distress. This suggests that a lack of suitable environments or options for the child's mental and physical growth causes stress among parents. Indeed, several studies have reported that independence was an important parental concern for a child with 22q11DS (Goodwin et al., 2020; Karas et al., 2014; Reilly et al., 2015).

A higher total number of challenges in medical and welfare areas in the full sample and educational area in the subsample were associated with increased psychological distress in parents. These associations remained significant even after adjusting for multimorbidity. Multiple challenges, including some items which were not statistically significant, were further associated with increased psychological distress.

This study has several implications. Parents—of children across all age intervals—who experienced medical, welfare, and educational challenges, and especially those who had multiple challenges, should be assessed for their mental health. Inadequate management by professionals and lack of support systems for 22q11DS were associated with parental psychological distress. This suggests that addressing challenges related to parental distress, rather than those frequently reported, needs to be focused on to support family mental health. Establishing a one‐stop medical care system and/or a multi‐disciplinary clinic for individuals with 22q11DS may improve parental mental health through better coordinated and comprehensive medical care for their children with 22q11DS. Promoting understanding about 22q11DS among the welfare staff and strengthening welfare support systems such as financial support and respite care may decrease parental psychological distress. Adequate management and provision of suitable services for the child's mental and physical development both inside and outside the educational settings can help to reduce psychological distress in parents.

We developed the survey with the assistance of clinical and research experts and parents from the concerned community. Since better research is produced when researchers and communities work together (Nature, 2018), we believe that this study provides valuable findings, not only for researchers but also for the concerned community. Despite these strengths, this study has some methodological limitations. First, the design of this study was cross‐sectional; thus, in future, longitudinal studies need to be conducted to address the causal relationships between the challenges and parental psychological distress. Second, since some items of the survey were our original creations, these might be less reliable. However, as mentioned in the Methods section, we performed a rigorous procedure to assess the validity of these items with the help of clinical and research experts and parents caring for an individual with 22q11DS. Third, there might be selection biases of the participants, because publicized population for the survey was unclear, and almost all participants (91.2%) were mothers. However, we adjusted the parental sex in all multivariate regression analyses. Fourth, we could not determine whether the findings are unique to 22q11DS, because it has not been compared to other genetic disorders. Fifth, we did not obtain certain potential covariates such as family status (number of parents and presence of sibling with 22q11DS or other disability) and distance from home to an available support organization. Finally, we did not control for the effect of parental diagnosis of 22q11DS. It is possible that parents and their child may share the challenges and that parental stress may be elevated due to parents' own challenges.

This study, to our knowledge, is the first and largest survey to explore the relationship between medical, social welfare, and educational challenges and psychological distress in parents caring for an individual with 22q11DS across a wide age range, and therefore, can provide a high potential for generalizability. The difficulty in going to multiple medical institutions, lack of systems or understanding among welfare staff, inadequate management in the classroom, and mismatch between service and users in educational settings were associated with significant parental psychological distress. Thus, to provide optimal care for families with a child with 22q11DS, it is important not only to assess the severity of comorbidities of the affected individual, but also to evaluate medical, welfare, and educational challenges perceived by parents, to support their mental health.

## CONFLICT OF INTEREST

The authors declare that they have no competing interests.

## AUTHOR CONTRIBUTIONS

Ryo Morishima, Yousuke Kumakura, Satoshi Usami, Akiko Kanehara, Miho Tanaka, Noriko Okochi, Naomi Nakajima, Junko Hamada, Tomoko Ogawa, Shuntaro Ando, Hidetaka Tamune, Mutsumi Nakahara, Seiichiro Jinde, Yukiko Kano, Kyoko Tanaka, Yoichiro Hirata, Akira Oka, and Kiyoto Kasai conceptualized and designed the study. Ryo Morishima, Yousuke Kumakura, Akiko Kanehara, Miho Tanaka, Noriko Okochi, Naomi Nakajima, Junko Hamada, Tomoko Ogawa, Hidetaka Tamune, Mutsumi Nakahara, Seiichiro Jinde, Yukiko Kano, Kyoko Tanaka, Yoichiro Hirata, Akira Oka, and Kiyoto Kasai acquired the data. Kiyoto Kasai acquired funding. Ryo Morishima and Satoshi Usami conducted the statistical analyses. Ryo Morishima wrote the first draft of the manuscript. All authors contributed to and have approved the final manuscript.

## Supporting information


**Table S1** Questionnaire about the medical, welfare, and educational challenges in the past year.

**Table S2** Psychiatric/neurological disorders of individuals with 22q11DS and Parental psychological distress (*N* = 125).

**Table S3** Hierarchical multivariable regression analysis on relationship between medical challenges and parental psychological distress (*N* = 125).

**Table S4** Hierarchical multivariable regression analysis on relationship between welfare challenges and parental psychological distress (*N* = 125).

**Table S5** Hierarchical multivariable regression analysis on relationship between educational challenges and parental psychological distress (*N* = 125).

**Table S6** Hierarchical multivariable regression analysis on relationship between medical challenges and parental psychological distress among subsamples (*N* = 84).

**Table S7** Hierarchical multivariable regression analysis on relationship between welfare challenges and parental psychological distress among subsamples (*N* = 84).

**Table S8** Hierarchical multivariable regression analysis on relationship between educational challenges and parental psychological distress among subsamples (*N* = 84).

## Data Availability

The data that support the findings of this study are available on request from the corresponding author. The data are not publicly available due to privacy or ethical restrictions.
