# Supplementary material for: Medical, welfare, and educational challenges and psychological distress in parents caring for an individual with 22q11.2 deletion syndrome: A cross‐sectional survey in Japan
Source: Am J Med Genet A. 2021 Sep 3;188(1):37–45. doi: 10.1002/ajmg.a.62485 (PMC9290134; doi:10.1002/ajmg.a.62485)
Supplement: Supplementary file 1 — Table S1 Questionnaire about the medical, welfare, and educational challenges in the past year. [file AJMG-188-37-s006.zip › AJMGA_62485_20210705_1_Supporting Information_TS1_1_22qChallenge_Morishima.docx]

| Table S1 Questionnaire about the medical, welfare, and educational challenges in the past year | |
| --- | --- |
| What challenges are you currently facing (in the past year) in terms of medical care as you support a family member with 22q11.2 deletion syndrome? Select all that apply. | |
| ⃞ | Lack of information regarding 22q11.2 deletion syndrome |
| ⃞ | Lack of knowledge on the part of medical staff (doctors, nurses, etc.) regarding 22q11.2 deletion syndrome |
| ⃞ | Attitude of medical staff |
| ⃞ | Lack of explanation from medical staff |
| ⃞ | Decision-making with regards to medical care |
| ⃞ | Informing the individual of their diagnosis with 22q11.2 deletion syndrome |
| ⃞ | Selecting a hospital for treatment |
| ⃞ | Difficulty of going to multiple medical institutions |
| ⃞ | Unable to receive comprehensive treatment due to multimorbidity |
| ⃞ | Difficulty selecting a hospital for mild diseases/symptoms |
| ⃞ | Individual with 22q11.2 deletion syndrome unable to undergo a consultation by his/herself |
| ⃞ | There are no medical institutions that allow parents to come for consultations if the individual with 22q11.2 deletion syndrome is unable to undergo a consultation |
| ⃞ | High medical expenses |
| ⃞ | Other ( ) |
