# Supplementary material for: Medical, welfare, and educational challenges and psychological distress in parents caring for an individual with 22q11.2 deletion syndrome: A cross‐sectional survey in Japan
Source: Am J Med Genet A. 2021 Sep 3;188(1):37–45. doi: 10.1002/ajmg.a.62485 (PMC9290134; doi:10.1002/ajmg.a.62485)
Supplement: Supplementary file 2 — Table S2 Psychiatric/neurological disorders of individuals with 22q11DS and Parental psychological distress (N = 125). [file AJMG-188-37-s004.docx]

| Table S2 Psychiatric/neurological disorders of individuals with 22q11DS and Parental psychological distress (N = 125) | | | | | | | | |
| --- | --- | --- | --- | --- | --- | --- | --- | --- |
|  |  |  | Parental psychological distress in Yes/No of each disorder of individuals with 22q11DS,  mean (sd) | | | |  |  |
|  | Yes, N | (%) | Yes | | No | | *t* | *p* |
| Schizophrenia | 8 | (6.4) | 9.4 | (4.4) | 4.7 | (4.5) | **2.87** | **0.005** |
| Depression | 1 | (0.8) | 17.0 | - | 4.9 | (4.5) | - | - |
| Bipolar disorder (manic depression) | 2 | (1.6) | 10.0 | (9.9) | 4.9 | (4.5) | 1.56 | 0.121 |
| Anxiety disorder | 13 | (10.4) | 8.7 | (6.2) | 4.5 | (4.2) | **2.37** | **0.034** |
| Panic disorder | 2 | (1.6) | 10.0 | (9.9) | 4.9 | (4.5) | 1.56 | 0.121 |
| Obsessive-compulsive disorder | 4 | (3.2) | 9.0 | (6.1) | 4.8 | (4.5) | 1.79 | 0.076 |
| Epilepsy | 19 | (15.2) | 7.0 | (6.3) | 4.6 | (4.2) | 1.54 | 0.138 |
| Parkinson’s disease | 0 | (0.0) | - | - | 5.0 | (4.6) | - | - |
| Other neuropsychiatric disorders | 7 | (5.6) | 5.0 | (3.3) | 5.0 | (4.7) | 0.01 | 0.989 |

Bold represents statistically significant.
