# Supplementary material for: Medical, welfare, and educational challenges and psychological distress in parents caring for an individual with 22q11.2 deletion syndrome: A cross‐sectional survey in Japan
Source: Am J Med Genet A. 2021 Sep 3;188(1):37–45. doi: 10.1002/ajmg.a.62485 (PMC9290134; doi:10.1002/ajmg.a.62485)
Supplement: Supplementary file 3 — Table S3 Hierarchical multivariable regression analysis on relationship between medical challenges and parental psychological distress (N = 125). [file AJMG-188-37-s008.docx]

| Table S3 Hierarchical multivariable regression analysis on relationship between medical challenges and parental psychological distress (N = 125) | | | | | | | | | | | | | | | | | | |
| --- | --- | --- | --- | --- | --- | --- | --- | --- | --- | --- | --- | --- | --- | --- | --- | --- | --- | --- |
|  |  |  |  |  | Crude model | | |  |  | Adjusted model 1 | | |  |  | Adjusted model 2 | | |  |
|  |  | Yes |  |  |  | 95%CI | |  |  |  | 95%CI | |  |  |  | 95%CI | |  |
|  |  | N | (%) |  | *β* | Low | Up | p |  | *β* | Low | Up | p |  | *β* | Low | Up | p |
|  | Total number of medical challenges, *mean (sd)* | *2.8* | *(2.0)* |  | **0.272** | **0.100** | **0.443** | **0.002** |  | **0.246** | **0.074** | **0.419** | **0.006** |  | **0.195** | **0.013** | **0.377** | **0.035** |
|  | Lack of information regarding 22q11.2 deletion syndrome | 77 | (61.6) |  | 0.003 | -0.175 | 0.182 | 0.973 |  |  |  |  |  |  |  |  |  |  |
|  | Lack of knowledge on the part of medical staff (doctors, nurses, etc.) regarding 22q11.2 deletion syndrome | 60 | (48.0) |  | 0.082 | -0.096 | 0.260 | 0.365 |  |  |  |  |  |  |  |  |  |  |
|  | Attitude of medical staff | 8 | (6.4) |  | **0.200** | **0.026** | **0.375** | **0.025** |  | 0.152 | -0.022 | 0.326 | 0.085 |  |  |  |  |  |
|  | Lack of explanation from medical staff | 15 | (12.0) |  | 0.034 | -0.144 | 0.212 | 0.706 |  |  |  |  |  |  |  |  |  |  |
|  | Decision-making with regards to medical care | 13 | (10.4) |  | 0.070 | -0.108 | 0.248 | 0.437 |  |  |  |  |  |  |  |  |  |  |
|  | Informing the individual of their diagnosis with 22q11.2 deletion syndrome | 22 | (17.6) |  | 0.103 | -0.075 | 0.280 | 0.253 |  |  |  |  |  |  |  |  |  |  |
|  | Selecting a hospital for treatment | 23 | (18.4) |  | **0.178** | **0.002** | **0.353** | **0.048** |  | 0.173 | -0.001 | 0.346 | 0.051 |  |  |  |  |  |
|  | Difficulty of going to multiple medical institutions | 32 | (25.6) |  | **0.238** | **0.065** | **0.412** | **0.007** |  | **0.214** | **0.046** | **0.382** | **0.013** |  | **0.180** | **0.012** | **0.349** | **0.036** |
|  | Unable to receive comprehensive treatment due to multimorbidity | 28 | (22.4) |  | **0.232** | **0.059** | **0.406** | **0.009** |  | 0.164 | -0.009 | 0.336 | 0.063 |  |  |  |  |  |
|  | Difficulty selecting a hospital for mild diseases/symptoms | 32 | (25.6) |  | 0.083 | -0.095 | 0.261 | 0.359 |  |  |  |  |  |  |  |  |  |  |
|  | Individual with 22q11.2 deletion syndrome unable to undergo a consultation by his/herself | 5 | (4.0) |  | 0.019 | -0.160 | 0.197 | 0.835 |  |  |  |  |  |  |  |  |  |  |
|  | There are no medical institutions that allow parents to come for consultations if the individual with 22q11.2 deletion syndrome is unable to undergo a consultation | 7 | (5.6) |  | 0.024 | -0.154 | 0.202 | 0.791 |  |  |  |  |  |  |  |  |  |  |
|  | High medical expenses | 5 | (4.0) |  | **0.196** | **0.021** | **0.371** | **0.028** |  | 0.172 | -0.001 | 0.345 | 0.051 |  |  |  |  |  |
|  | Other | 19 | (15.2) |  | 0.031 | -0.147 | 0.210 | 0.729 |  |  |  |  |  |  |  |  |  |  |
| β, standardized regression coefficient; CI, confidence interval. Bold represents statistically significant. | | | | | | | | | | | |  |  |  |  |  |  |  |
| Crude model: simple regression analysis. | |  |  |  |  |  |  |  |  |  |  |  |  |  |  |  |  |  |
| Adjusted model 1: multivariate regression analysis adjusting parental age, parental sex, family income, marital status, child age, and child sex. | | | | | | | | | | | | | | | | | | |
| Adjusted model 2: multivariate regression analysis adjusting parental age, parental sex, family income, marital status, child age, child sex, and total number of child’s comorbidities. | | | | | | | | | | | | | | | | | | |
