# Supplementary material for: Medical, welfare, and educational challenges and psychological distress in parents caring for an individual with 22q11.2 deletion syndrome: A cross‐sectional survey in Japan
Source: Am J Med Genet A. 2021 Sep 3;188(1):37–45. doi: 10.1002/ajmg.a.62485 (PMC9290134; doi:10.1002/ajmg.a.62485)
Supplement: Supplementary file 4 — Table S4 Hierarchical multivariable regression analysis on relationship between welfare challenges and parental psychological distress (N = 125). [file AJMG-188-37-s003.docx]

| Table S4 Hierarchical multivariable regression analysis on relationship between welfare challenges and parental psychological distress (N = 125) | | | | | | | | | | | | | | | | | | |
| --- | --- | --- | --- | --- | --- | --- | --- | --- | --- | --- | --- | --- | --- | --- | --- | --- | --- | --- |
|  |  |  |  |  | Crude model | | |  |  | Adjusted model 1 | | |  |  | Adjusted model 2 | | |  |
|  |  | Yes |  |  |  | 95%CI | |  |  |  | 95%CI | |  |  |  | 95%CI | |  |
|  |  | N | (%) |  | *β* | Low | Up | p |  | *β* | Low | Up | p |  | *β* | Low | Up | p |
|  | Total number of welfare challenges, *mean (sd)* | *5.3* | *(4.4)* |  | **0.300** | **0.130** | **0.470** | **0.001** |  | **0.298** | **0.125** | **0.471** | **0.001** |  | **0.264** | **0.090** | **0.438** | **0.003** |
|  | Lack of information on financial support systems | 39 | (31.2) |  | 0.086 | -0.092 | 0.264 | 0.340 |  |  |  |  |  |  |  |  |  |  |
|  | Lack of consultants and contacts regarding financial support systems | 25 | (20.0) |  | **0.203** | **0.028** | **0.377** | **0.023** |  | **0.251** | **0.081** | **0.421** | **0.004** |  | **0.237** | **0.069** | **0.404** | **0.006** |
|  | Lack of financial support systems | 29 | (23.2) |  | 0.102 | -0.076 | 0.279 | 0.259 |  |  |  |  |  |  |  |  |  |  |
|  | In spite of the fact that three disabilities are overlapping (physical, intellectual, and mental), the system (disability certificate, disability pension, etc.) is divided vertically into physical disabilities, intellectual disabilities, and mental disabilities, and the difficulty of overlapping disorders when filing applications has not been taken into consideration | 43 | (34.4) |  | 0.172 | -0.004 | 0.348 | 0.055 |  |  |  |  |  |  |  |  |  |  |
|  | Unable to receive intractable disease certification | 22 | (17.6) |  | 0.103 | -0.075 | 0.280 | 0.253 |  |  |  |  |  |  |  |  |  |  |
|  | Lack of information regarding development-related support such as rehabilitation | 55 | (44.0) |  | -0.037 | -0.216 | 0.141 | 0.679 |  |  |  |  |  |  |  |  |  |  |
|  | Lack of consultants and contacts regarding development-related support such as rehabilitation | 36 | (28.8) |  | 0.130 | -0.047 | 0.307 | 0.148 |  |  |  |  |  |  |  |  |  |  |
|  | Lack of development-related support | 34 | (27.2) |  | 0.038 | -0.140 | 0.217 | 0.671 |  |  |  |  |  |  |  |  |  |  |
|  | Lack of information on welfare services | 41 | (32.8) |  | 0.085 | -0.093 | 0.263 | 0.345 |  |  |  |  |  |  |  |  |  |  |
|  | Lack of consultants and contacts regarding welfare services | 25 | (20.0) |  | 0.003 | -0.176 | 0.181 | 0.977 |  |  |  |  |  |  |  |  |  |  |
|  | Lack of knowledge on the part of supporters (welfare facility personnel and government personnel) regarding 22q11.2 deletion syndrome | 60 | (48.0) |  | **0.298** | **0.127** | **0.468** | **0.001** |  | **0.284** | **0.113** | **0.455** | **0.001** |  | **0.259** | **0.089** | **0.429** | **0.003** |
|  | No daycare center suitable for the characteristics and traits of the individual with 22q11.2 deletion syndrome | 31 | (24.8) |  | **0.341** | **0.174** | **0.509** | **0.000** |  | **0.294** | **0.124** | **0.465** | **0.001** |  | **0.268** | **0.098** | **0.438** | **0.002** |
|  | Unable to go to the daycare center even if one is available | 12 | (9.6) |  | **0.203** | **0.028** | **0.377** | **0.024** |  | **0.203** | **0.026** | **0.380** | **0.025** |  | 0.162 | -0.017 | 0.341 | 0.075 |
|  | Lack of home care/visiting services | 13 | (10.4) |  | **0.184** | **0.009** | **0.360** | **0.040** |  | 0.169 | -0.014 | 0.352 | 0.070 |  |  |  |  |  |
|  | No support available when parents are exhausted and need a respite | 25 | (20.0) |  | **0.320** | **0.151** | **0.489** | **0.000** |  | **0.352** | **0.191** | **0.514** | **0.000** |  | **0.322** | **0.159** | **0.486** | **0.000** |
|  | Lack of information about employment | 29 | (23.2) |  | 0.139 | -0.038 | 0.316 | 0.122 |  |  |  |  |  |  |  |  |  |  |
|  | Lack of consultants and contacts regarding employment support | 16 | (12.8) |  | 0.158 | -0.018 | 0.334 | 0.078 |  |  |  |  |  |  |  |  |  |  |
|  | No job opportunities | 8 | (6.4) |  | **0.236** | **0.063** | **0.409** | **0.008** |  | 0.162 | -0.023 | 0.348 | 0.086 |  |  |  |  |  |
|  | Unable to remain employed for extended periods | 3 | (2.4) |  | 0.092 | -0.086 | 0.269 | 0.309 |  |  |  |  |  |  |  |  |  |  |
|  | Lack of understanding in the workplace | 6 | (4.8) |  | **0.319** | **0.149** | **0.488** | **0.000** |  | **0.264** | **0.089** | **0.439** | **0.003** |  | **0.239** | **0.065** | **0.413** | **0.008** |
|  | Lack of help / support in daily life | 13 | (10.4) |  | -0.004 | -0.182 | 0.175 | 0.965 |  |  |  |  |  |  |  |  |  |  |
|  | Lack of residences such as group homes | 14 | (11.2) |  | **0.322** | **0.153** | **0.491** | **0.000** |  | **0.194** | **0.005** | **0.384** | **0.045** |  | 0.161 | -0.028 | 0.350 | 0.094 |
|  | Concerns about marriage | 28 | (22.4) |  | 0.111 | -0.066 | 0.289 | 0.217 |  |  |  |  |  |  |  |  |  |  |
|  | Concerns about pregnancy and childbirth | 18 | (14.4) |  | 0.022 | -0.156 | 0.200 | 0.808 |  |  |  |  |  |  |  |  |  |  |
|  | Individual with 22q11.2 deletion syndrome cannot enroll in life insurance | 26 | (20.8) |  | 0.067 | -0.111 | 0.245 | 0.458 |  |  |  |  |  |  |  |  |  |  |
|  | Other | 13 | (10.4) |  | -0.095 | -0.273 | 0.083 | 0.291 |  |  |  |  |  |  |  |  |  |  |
| β, standardized regression coefficient; CI, confidence interval. Bold represents statistically significant. | | | | | | | | | | | |  |  |  |  |  |  |  |
| Crude model: simple regression analysis. | |  |  |  |  |  |  |  |  |  |  |  |  |  |  |  |  |  |
| Adjusted model 1: multivariate regression analysis adjusting parental age, parental sex, family income, marital status, child age, and child sex. | | | | | | | | | | | | | | | | | | |
| Adjusted model 2: multivariate regression analysis adjusting parental age, parental sex, family income, marital status, child age, child sex, and total number of child’s comorbidities. | | | | | | | | | | | | | | | | | | |
