# Supplementary material for: Medical, welfare, and educational challenges and psychological distress in parents caring for an individual with 22q11.2 deletion syndrome: A cross‐sectional survey in Japan
Source: Am J Med Genet A. 2021 Sep 3;188(1):37–45. doi: 10.1002/ajmg.a.62485 (PMC9290134; doi:10.1002/ajmg.a.62485)
Supplement: Supplementary file 6 — Table S6 Hierarchical multivariable regression analysis on relationship between medical challenges and parental psychological distress among subsamples (N = 84). [file AJMG-188-37-s001.docx]

| Table S6 Hierarchical multivariable regression analysis on relationship between medical challenges and parental psychological distress amongst subsamples (N = 84) | | | | | | | | | | | | | | | | | | |
| --- | --- | --- | --- | --- | --- | --- | --- | --- | --- | --- | --- | --- | --- | --- | --- | --- | --- | --- |
|  |  |  |  |  | Crude model | | |  |  | Adjusted model 1 | | |  |  | Adjusted model 2 | | |  |
|  |  | Yes |  |  |  | 95%CI | |  |  |  | 95%CI | |  |  |  | 95%CI | |  |
|  |  | N | (%) |  | *β* | Low | Up | p |  | *β* | Low | Up | p |  | *β* | Low | Up | p |
|  | Total number of medical challenges, *mean (sd)* | *2.4* | *(1.7)* |  | **0.231** | **0.017** | **0.445** | **0.035** |  | 0.197 | -0.036 | 0.430 | 0.097 |  |  |  |  |  |
|  | Lack of information regarding 22q11.2 deletion syndrome | 50 | (59.5) |  | 0.039 | -0.181 | 0.258 | 0.726 |  |  |  |  |  |  |  |  |  |  |
|  | Lack of knowledge on the part of medical staff (doctors, nurses, etc.) regarding 22q11.2 deletion syndrome | 35 | (41.7) |  | 0.110 | -0.108 | 0.329 | 0.318 |  |  |  |  |  |  |  |  |  |  |
|  | Attitude of medical staff | 4 | (4.8) |  | 0.127 | -0.091 | 0.345 | 0.248 |  |  |  |  |  |  |  |  |  |  |
|  | Lack of explanation from medical staff | 9 | (10.7) |  | -0.003 | -0.223 | 0.216 | 0.977 |  |  |  |  |  |  |  |  |  |  |
|  | Decision-making with regards to medical care | 6 | (7.1) |  | 0.116 | -0.102 | 0.335 | 0.292 |  |  |  |  |  |  |  |  |  |  |
|  | Informing the individual of their diagnosis with 22q11.2 deletion syndrome | 14 | (16.7) |  | -0.004 | -0.224 | 0.216 | 0.97 |  |  |  |  |  |  |  |  |  |  |
|  | Selecting a hospital for treatment | 12 | (14.3) |  | 0.093 | -0.126 | 0.311 | 0.402 |  |  |  |  |  |  |  |  |  |  |
|  | Difficulty of going to multiple medical institutions | 18 | (21.4) |  | **0.301** | **0.092** | **0.511** | **0.005** |  | **0.293** | **0.073** | **0.514** | **0.010** |  | **0.273** | **0.048** | **0.499** | **0.018** |
|  | Unable to receive comprehensive treatment due to multimorbidity | 12 | (14.3) |  | **0.250** | **0.037** | **0.463** | **0.022** |  | 0.207 | -0.014 | 0.429 | 0.067 |  |  |  |  |  |
|  | Difficulty selecting a hospital for mild diseases/symptoms | 20 | (23.8) |  | -0.027 | -0.246 | 0.193 | 0.809 |  |  |  |  |  |  |  |  |  |  |
|  | Individual with 22q11.2 deletion syndrome unable to undergo a consultation by his/herself | 0 | (0.0) |  | - | - | - | - |  |  |  |  |  |  |  |  |  |  |
|  | There are no medical institutions that allow parents to come for consultations if the individual with 22q11.2 deletion syndrome is unable to undergo a consultation | 5 | (6.0) |  | 0.075 | -0.144 | 0.294 | 0.496 |  |  |  |  |  |  |  |  |  |  |
|  | High medical expenses | 2 | (2.4) |  | 0.139 | -0.078 | 0.357 | 0.207 |  |  |  |  |  |  |  |  |  |  |
|  | Other | 10 | (11.9) |  | -0.051 | -0.270 | 0.169 | 0.647 |  |  |  |  |  |  |  |  |  |  |
| β, standardized regression coefficient; CI, confidence interval. Bold represents statistically significant. | | | | | | | | | | | |  |  |  |  |  |  |  |
| Crude model: simple regression analysis. | |  |  |  |  |  |  |  |  |  |  |  |  |  |  |  |  |  |
| Adjusted model 1: multivariate regression analysis adjusting parental age, parental sex, family income, marital status, child age, and child sex. | | | | | | | | | | | | | | | | | | |
| Adjusted model 2: multivariate regression analysis adjusting parental age, parental sex, family income, marital status, child age, child sex, and total number of child’s comorbidities. | | | | | | | | | | | | | | | | | | |
