# Supplementary material for: Medical, welfare, and educational challenges and psychological distress in parents caring for an individual with 22q11.2 deletion syndrome: A cross‐sectional survey in Japan
Source: Am J Med Genet A. 2021 Sep 3;188(1):37–45. doi: 10.1002/ajmg.a.62485 (PMC9290134; doi:10.1002/ajmg.a.62485)
Supplement: Supplementary file 8 — Table S8 Hierarchical multivariable regression analysis on relationship between educational challenges and parental psychological distress among subsamples (N = 84). [file AJMG-188-37-s005.docx]

| Table S8 Hierarchical multivariable regression analysis on relationship between educational challenges and parental psychological distress amongst subsamples (N = 84) | | | | | | | | | | | | | | | | | | |
| --- | --- | --- | --- | --- | --- | --- | --- | --- | --- | --- | --- | --- | --- | --- | --- | --- | --- | --- |
|  |  |  |  |  | Crude model | | |  |  | Adjusted model 1 | | |  |  | Adjusted model 2 | | |  |
|  |  | Yes |  |  |  | 95%CI | |  |  |  | 95%CI | |  |  |  | 95%CI | |  |
|  |  | N | (%) |  | *β* | Low | Up | p |  | *β* | Low | Up | p |  | *β* | Low | Up | p |
|  | Total number of educational challenges, *mean (sd)* | *3.3* | *(3.1)* |  | **0.318** | **0.110** | **0.526** | **0.003** |  | **0.288** | **0.066** | **0.510** | **0.012** |  | **0.269** | **0.043** | **0.495** | **0.020** |
|  | Preschool / kindergarten staff were unhelpful | 2 | (2.4) |  | -0.162 | -0.379 | 0.055 | 0.141 |  |  |  |  |  |  |  |  |  |  |
|  | Reluctance to attending kindergarten or preschool | 3 | (3.6) |  | -0.002 | -0.221 | 0.218 | 0.987 |  |  |  |  |  |  |  |  |  |  |
|  | Lack of information regarding school selection | 19 | (22.6) |  | 0.017 | -0.203 | 0.237 | 0.878 |  |  |  |  |  |  |  |  |  |  |
|  | Lack of consultants or contacts regarding school selection | 15 | (17.9) |  | -0.012 | -0.232 | 0.207 | 0.912 |  |  |  |  |  |  |  |  |  |  |
|  | No educational institutions suitable for the individual’s characteristics / traits | 18 | (21.4) |  | **0.264** | **0.052** | **0.476** | **0.015** |  | **0.239** | **0.014** | **0.465** | **0.038** |  | **0.227** | **0.001** | **0.453** | **0.049** |
|  | Lack of special classes / schools for special needs education | 12 | (14.3) |  | -0.083 | -0.301 | 0.136 | 0.455 |  |  |  |  |  |  |  |  |  |  |
|  | Lack of institutions for higher education that the individual could go to | 11 | (13.1) |  | 0.096 | -0.122 | 0.315 | 0.383 |  |  |  |  |  |  |  |  |  |  |
|  | Lack of home / visiting educational services | 2 | (2.4) |  | 0.099 | -0.120 | 0.318 | 0.37 |  |  |  |  |  |  |  |  |  |  |
|  | The regular class teachers were unhelpful | 6 | (7.1) |  | **0.319** | **0.110** | **0.527** | **0.003** |  | **0.303** | **0.085** | **0.521** | **0.007** |  | **0.292** | **0.074** | **0.510** | **0.009** |
|  | The special class teachers were unhelpful | 7 | (8.3) |  | **0.274** | **0.063** | **0.486** | **0.012** |  | **0.287** | **0.058** | **0.515** | **0.015** |  | **0.270** | **0.040** | **0.500** | **0.022** |
|  | The schools for special needs education teachers were unhelpful | 1 | (1.2) |  | -0.086 | -0.305 | 0.133 | 0.438 |  |  |  |  |  |  |  |  |  |  |
|  | Cannot keep up with school work | 18 | (21.4) |  | 0.182 | -0.034 | 0.398 | 0.0979 |  |  |  |  |  |  |  |  |  |  |
|  | Difficult to participate in events such as field days and school festivals | 5 | (6.0) |  | -0.145 | -0.362 | 0.073 | 0.189 |  |  |  |  |  |  |  |  |  |  |
|  | Difficulty making friends | 12 | (14.3) |  | 0.145 | -0.072 | 0.362 | 0.188 |  |  |  |  |  |  |  |  |  |  |
|  | Refusal to attend school | 5 | (6.0) |  | **0.270** | **0.058** | **0.481** | **0.013** |  | **0.308** | **0.072** | **0.544** | **0.011** |  | **0.292** | **0.054** | **0.530** | **0.017** |
|  | Being bullied | 5 | (6.0) |  | 0.075 | -0.144 | 0.294 | 0.496 |  |  |  |  |  |  |  |  |  |  |
|  | What to do after school | 17 | (20.2) |  | 0.171 | -0.046 | 0.387 | 0.121 |  |  |  |  |  |  |  |  |  |  |
|  | Participating in extracurricular lessons and activities | 9 | (10.7) |  | **0.324** | **0.116** | **0.531** | **0.003** |  | **0.345** | **0.128** | **0.562** | **0.002** |  | **0.338** | **0.122** | **0.555** | **0.003** |
|  | Communicating with the home room teacher | 9 | (10.7) |  | **0.304** | **0.095** | **0.513** | **0.005** |  | **0.283** | **0.057** | **0.508** | **0.015** |  | **0.261** | **0.030** | **0.492** | **0.027** |
|  | Change of home room teacher | 6 | (7.1) |  | **0.307** | **0.098** | **0.516** | **0.005** |  | **0.307** | **0.085** | **0.529** | **0.007** |  | **0.289** | **0.050** | **0.528** | **0.019** |
|  | Lack of knowledge regarding 22q11.2 deletion syndrome on the part of school staff (faculty, etc.) | 34 | (40.5) |  | 0.155 | -0.062 | 0.372 | 0.160 |  |  |  |  |  |  |  |  |  |  |
|  | Lack of understanding on the part of school staff (faculty, etc.) of the fact that children with 22q11.2 deletion syndrome may need more individual attention than children with ordinary physical or intellectual disabilities | 27 | (32.1) |  | **0.263** | **0.051** | **0.475** | **0.016** |  | 0.220 | -0.004 | 0.445 | 0.054 |  |  |  |  |  |
|  | Cannot come to an agreement with the school regarding educational policy for the individual with 22q11.2 deletion syndrome | 5 | (6.0) |  | 0.127 | -0.091 | 0.345 | 0.249 |  |  |  |  |  |  |  |  |  |  |
|  | You (the parent or guardian) or your family’s anxiety or resistance to selecting a special class or schools for special needs education | 13 | (15.5) |  | 0.047 | -0.173 | 0.266 | 0.672 |  |  |  |  |  |  |  |  |  |  |
|  | Anxiety or resistance that the individual feels on selecting a special class or schools for special needs education | 4 | (4.8) |  | 0.099 | -0.120 | 0.317 | 0.372 |  |  |  |  |  |  |  |  |  |  |
|  | Other | 10 | (11.9) |  | -0.193 | -0.408 | 0.023 | 0.079 |  |  |  |  |  |  |  |  |  |  |
| β, standardized regression coefficient; CI, confidence interval. Bold represents statistically significant. | | | | | | | | | | | | | |  |  |  |  |  |
| Crude model: simple regression analysis. | |  |  |  |  |  |  |  |  |  |  |  |  |  |  |  |  |  |
| Adjusted model 1: multivariate regression analysis adjusting parental age, parental sex, family income, marital status, child age, and child sex. | | | | | | | | | | | | | | | | | | |
| Adjusted model 2: multivariate regression analysis adjusting parental age, parental sex, family income, marital status, child age, child sex, and total number of child’s comorbidities. | | | | | | | | | | | | | | | | | | |
